# A Caulobacter crescentus Microbicide Protects from Vaginal Infection with HIV-1_JR-CSF_ in Humanized Bone Marrow-Liver-Thymus Mice

**DOI:** 10.1128/JVI.00614-19

**Published:** 2019-08-28

**Authors:** Christina Farr Zuend, John F. Nomellini, John Smit, Marc S. Horwitz

**Affiliations:** a Department of Microbiology and Immunology, University of British Columbia, Vancouver, British Columbia, Canada; Icahn School of Medicine at Mount Sinai

**Keywords:** *Caulobacter crescentus*, HIV, microbicide, humanized mice

## Abstract

Over 2 million people are infected with HIV-1 annually. Approximately half of these new infections occur in women residing in low-income countries, where their access to and control over HIV-1 preventative measures are often limited, indicating that female-controlled prevention options for HIV-1 are urgently needed. Microbicides that can be topically applied to the vaginal tract in advance of sexual activity represent a promising female-controlled prevention option for HIV-1. We have previously described the development of an HIV-1-specific microbicide using the surface or S-layer recombinant protein display capabilities of the nonpathogenic, freshwater bacterium Caulobacter crescentus. Recombinant C. crescentus bacteria were created that displayed proteins that interfere with the HIV-1 attachment and entry process and that were able to provide significant protection of TZM-bl cells from infection with HIV-1 pseudovirus. These studies have been expanded to investigate if these recombinant C. crescentus bacteria are able to maintain efficacy with replication-competent HIV-1 and both TZM-bl cells and human peripheral blood mononuclear cells (PBMCs). In addition, we utilized the humanized bone marrow-liver-thymus (BLT) mouse model to determine if vaginal application of recombinant C. crescentus at the time of HIV-1_JR-CSF_ infection could provide protection from HIV-1 infection. Recombinant C. crescentus bacteria expressing Griffithsin, GB virus C E2 protein, elafin, α-1-antitrypsin, indolicidin, and the fusion inhibitor T-1249 were able to protect 40 to 75% of the BLT mice from vaginal infection with HIV-1_JR-CSF_, with C. crescentus bacteria expressing Griffithsin being the most effective. Taken together, these data suggest that a C. crescentus-based microbicide could be a safe and effective method for HIV-1 prevention.

**IMPORTANCE** Human immunodeficiency virus (HIV) disproportionally infects young women in sub-Saharan Africa. Current HIV-1 prevention options have had limited success among women, suggesting that alternative, female-controlled prevention options need to be developed. Microbicides that can be applied to the vaginal tract are a promising prevention option. In this study, we describe the testing of 15 potential candidates for inhibition of HIV-1 infection in a humanized mouse model of HIV-1 infection. Four of these candidates were able to provide significant protection from vaginal infection with HIV-1, with the most successful candidate protecting 75% of the mice from infection. This study describes the preclinical testing of a new strategy that could be a safe and effective option for HIV-1 prevention in women.

## Abstract (precis)

Human immunodeficiency virus (HIV) disproportionally infects young women in sub-Saharan Africa. Current HIV-1 prevention options have had limited success among women, suggesting that alternative, female-controlled prevention options need to be developed. Microbicides that can be applied to the vaginal tract are a promising prevention option. In this study, we describe the testing of 15 potential candidates for inhibition of HIV-1 infection in a humanized mouse model of HIV-1 infection. Four of these candidates were able to provide significant protection from vaginal infection with HIV-1, with the most successful candidate protecting 75% of the mice from infection. This study describes the preclinical testing of a new strategy that could be a safe and effective option for HIV-1 prevention in women.

## INTRODUCTION

HIV-1 prevention for women is a global health priority. Approximately 1 million women are infected with HIV-1 each year (1). In sub-Saharan Africa, 75% of new HIV-1 infections in 15- to 19-year-olds are among women, making them twice as likely to acquire HIV-1 infection as men (1, 2). Existing HIV-1 prevention options, like condoms, male circumcision, and viral suppression, are not always feasible for women, as these rely on their partners for use. Thus, prevention options that women can use and control are urgently needed. With the difficulties in developing an effective vaccine and the inconsistent results of preexposure prophylaxis (PrEP) in women (1, 3–5), alternative female-controlled HIV-1 prevention options are urgently needed.

Recent microbicide clinical trials have focused on either a 1% tenofovir gel or a dapivirine vaginal ring for HIV-1 prevention (2, 6–8). Tenofovir gel demonstrated 39% efficacy in the CAPRISA 004 clinical trial (2). However, in both the FACTS 001 and VOICE trials, it had no protective effect on HIV-1 acquisition (2–4, 9). While adherence was a concern, follow-up studies have indicated that vaginal microbiota, particularly *Gardnerella* and *Prevotella*, can interfere with the effectiveness of tenofovir-based microbicides (10). In the ASPIRE and The Ring Study trials, the dapivirine vaginal ring reduced HIV-1 acquisition by 27% and 31%, respectively (7, 8). However, among women under the age of 21 years, the ring was 15% effective in The Ring Study and had no efficacy in the ASPIRE trial (7, 8). These clinical trial results indicate that the development of alternative microbicide strategies is urgently needed.

We have previously demonstrated up to 72% protection from HIV-1 infection *in vitro* using the surface or S-layer recombinant display capabilities of the nonpathogenic, freshwater bacterium Caulobacter crescentus (11–13). In these studies, 15 unique recombinant C. crescentus bacteria with the ability to prevent the attachment or entry of HIV-1 into a target cell were created (11, 12). The recombinant bacteria expressed a wide variety of anti-HIV proteins, including the carbohydrate binding agents cyanovirin-N (14), microvirin (15), and griffithsin (16, 17), ligands (macrophage inflammatory protein 1α [MIP-1α]) (18), decoy receptors (CD4, mimetic CD4M33F23) (18, 19), fusion inhibitors (Fuzeon [20], T-1249 [21], C52 variant [22]), and the antimicrobial peptides BmKn2 (23), α-1-antitrypsin (A1AT) (24), indolicidin (25), and elafin (26). The success of these recombinants for HIV-1 prevention in initial studies indicated that further studies using more physiologically relevant models are warranted.

While C. crescentus is a nonpathogenic bacterium, it is a Gram-negative bacterium that could stimulate an immune response *in vivo*. Previous work in our lab (13) and by collaborators (27) has demonstrated that C. crescentus appears to be safe for topical application to the vaginal tract. Importantly, there was no significant production of inflammatory cytokines, immune cell recruitment, or antibody production after vaginal application of C. crescentus in an immunocompetent mouse model (13). Furthermore, C. crescentus cannot be cultured from the peritoneal cavity of immunocompetent mice within 10 days following intraperitoneal injection (27). These data suggest that C. crescentus will likely be safe for use as a topical mucosal agent.

Herein, both *in vitro* and *in vivo* studies were undertaken to further test the ability of recombinant C. crescentus to prevent HIV-1 infection. *In vitro* studies using replication-competent HIV-1 isolates from several strains indicated that both TZM-bl cells and human peripheral blood mononuclear cells (PBMCs) were protected from HIV-1 infection. In addition, the recombinant C. crescentus was applied to the vaginal tract of humanized bone marrow-liver-thymus (BLT) mice (28–30), and HIV-1 infection was measured. We found that 40 to 75% of mice were protected from vaginal infection with HIV-1 using 6 different recombinants, with the C. crescentus recombinant expressing griffithsin (Cc-griffithsin) being the most effective at preventing HIV-1 acquisition. Taken together, these data suggest that a C. crescentus-based microbicide could be a worthwhile option for developing a safe and effective method for HIV-1 prevention.

## RESULTS

### Many recombinant C. crescentus bacteria provide protection from replication-competent HIV-1 infection.

**FIG 1:**
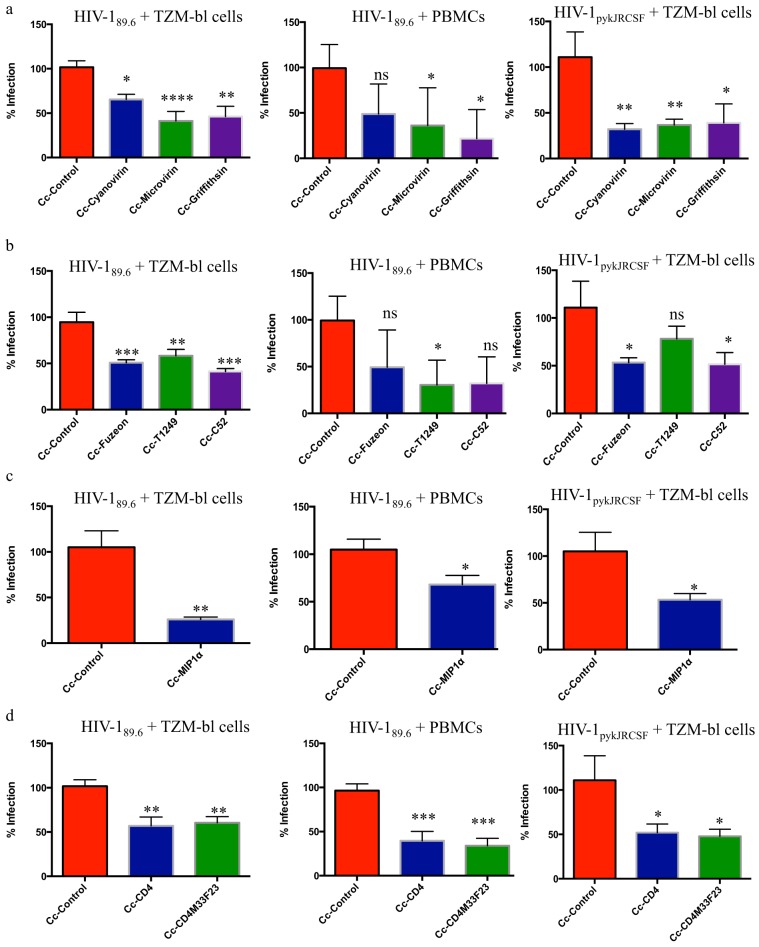
Viral blocking assays. A total of 10,000 TZM-bl cells or PHA-stimulated PBMCs were incubated for 48 to 72 h with 200 TCID_50_ of HIV-1_89.6_ or HIV-1_pyk-JR-CSF_ and 10^8^ recombinant C. crescentus bacteria. HIV-1 infection was measured using a β-galactosidase assay (TZM-bl cells) or p24 ELISA (PBMCs). To minimize assay-to-assay variability, the wells containing cells and HIV-1 were set as 100% infection, and the results for the other wells were normalized to the 100% infection. Each experiment was performed in quadruplicate and repeated three times. An unpaired two-tailed *t* test (for the Cc-control group and 1 group receiving recombinant C. crescentus bacteria) or ANOVA with Bonferroni’s correction for multiple comparisons (for the Cc-control group and >2 groups receiving recombinant C. crescentus bacteria, with each group being compared to the Cc-control group and each other) were used to evaluate the significance of differences between groups as appropriate. Results are reported as the mean + SEM. (a) Antiviral lectins. (b) Fusion inhibitors. (c) MIP-1α. (d) CD4-based inhibitors. *, *P* < 0.05; **, *P* < 0.01; ***, *P* < 0.001; ****, *P* < 0.0001; ns, not significant (*P* > 0.05).

We have previously demonstrated a successful proof of concept for a recombinant C. crescentus-based microbicide expressing antiviral lectins, fusion inhibitors, decoys, and antiviral peptides (11–13), demonstrating inhibition of infection using HIV-1 pseudoviruses representing the two most common viral clades, B and C. These studies were repeated using replication-competent HIV-1 and both TZM-bl cells and human PBMCs. Previous studies have indicated that 1 × 10^8^ recombinant bacteria provide the best protection from HIV-1 infection while minimizing nonspecific inhibition by control (no insert) C. crescentus bacteria (Cc-control) (13). HIV-1 infection rates were significantly decreased with all recombinant bacteria across various HIV-1 strains in both cell types. While the most effective recombinant varied depending on the virus/cell combination, several recombinants were able to provide a >90% decrease in HIV-1 infection in both TZM-bl cells and PBMCs with specific viral strains (Table 1; Fig. 1). With some of the recombinants, there was a wide range of effectiveness depending on the viral strain used, which is why we anticipate combining recombinants to improve efficacy in a final microbicide product. Notably, HIV-1 was suppressed, on average, by 50% with each recombinant, results that were consistent with those of our previous studies.

**TABLE 1:** Recombinant C. crescentus

Protein	Name	Sequence	Reference in which construct was first described	Mean (range) % HIV-1 inhibition	
				Live virus-infected TZM-bl cells^*a*^	Live virus-infected PBMCs^*b*^
Blank control	Cc-control	NA^*c*^	11	110.1 (80.8–137.7)^*d*^	100 (99.9–100)^*d*^
Griffithsin	Cc-griffithsin	SLTHRKFGGSGGSPFSGLSSIAVRSGSYLDAIIIDGVHHGGSGGNLSPTFTFGSGEYISNMTIRSGDYIDNISFETNMGRRFGPYGGSGGSANTLSNVKVIQINGSAGDYLDSLDIYYEQY	12	61 (19.6–85.2)	56.7 (25.4–100)
Microvirin	Cc-microvirin	MPNFSHTCSSINYDPDSTILSAECQARDGEWLPTELRLSDHIGNIDGELQFGDQNFQETCQDCHLEFGDGEQSVWLVCTCQTMDGEWKSTQILLDSQIDNNDSQLEIG	12	61.8 (11.9–82.4)	56 (−7.9–100)
Cyanovirin	Cc-cyanovirin	LGKFSQTCYNSAIQGSVLTSTCERTNGGYNTSSIDLNSVIENVDGSLKWQGSNFIETCRNTQLAGSSELAAECKTRAQQFVSTKINLDDHIANIDGTLKYE	12	51.6 (21.6–87.6)	71.6 (15.9–100)
Fuzeon, T-20	Cc-Fuzeon	MYTSLIHSLIEESQNQQEKNEQELLELDKWASLWNWFM	12	58.8 (29.3–78.9)	62 (8.3–100)
T-1249	Cc-T1249	WQEWEQKITALLEQAQIQQEKNEYELQKLDKWASLWEWF	12	40 (4.9–54.2)	27.6 (−30.9–100)
C52	Cc-C52	NHTTWMEWDREINNYTSLIHSLIEESQNQQEKNEQELLELDKWASLWNWFNI	12	50.7 (12.2–81.3)	86.8 (46.5–100)
MIP-1α	Cc-MIP1α	APLAADTPTACCFSYTSRQIPQNFIADYFETSSQCSKPSVIFLTKRGRQVCADPSEEWVQKYVSDLELSA	11	60.3 (23–83)	69.2 (11.2–97.2)
CD4	Cc-CD4	GDTVELTCTASQKKSIQFHWKNSNQIKILGNQGSFLTKGPSKLNDRADSRRSLWDQGNFPLIIKNLKIEDSDTYICEVEDQ	11	55.5 (10.6–72.5)	74.6 (18–97.5)
CD4 mimetic	Cc-CD4M33F23	NLHFCQLRCKSLGLLGKCAGSFCACV	12	36.2 (16–74.3)	26.9 (36.5–93.2)
BmKn2	Cc-BmKn2	FIGAIARLLSKIF	—^*e*^	—	—
α-1-antitrypsin	Cc-A1AT	LEAIPCSIPPEFLFGKPFVFLMIEQNTKSPLFMG	—	—	—
Elafin	Cc-elafin	AQEPVKGPVSTKPGSCPIILIRCAMLNPPNRCLKDTDCPGIKKCCEGSCGMACFVPQ	—	—	—
Indolicidin	Cc-indolicidin	ILPWKWPWWPWRR	—	—	—
GB Virus C E2 protein	Cc-GBVCE2	WDRGNVTLLCDCPNGPWVWVPAFCQAVG	Herein	64.9 (19.5–86.4)	75.7 (27.2–87.9)

a The mean and range presented are a summary of data from HIV-1 strains 89.6, BaL, JR-FL, and pykJR-CSF.

b The mean and range presented are a summary of data from HIV-1 strains 89.6, JR-FL, and SF162.

*^c^*NA, not applicable.

d Data represent the mean (range) percent infection.

e —, data are presented elsewhere (13).

### GB virus C E2 protein blocks HIV-1 infection *in vitro*.

**FIG 2:**
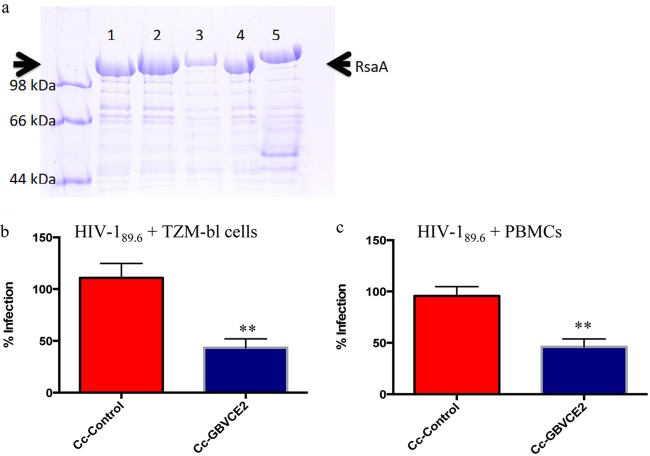
Cc-GBVCE2 provides protection from HIV-1 infection. (a) Coomassie blue-stained 7.5% SDS-PAGE gel of normalized low-pH-extracted RsaA protein from C. crescentus strain JS 4038 containing RsaA plasmids. Arrows indicate the location of the S-layer protein. Lane 1, Cc-control; lane 2, Cc-GBVCE2; lanes 3 to 5, S-layer expression controls. (b and c) A total of 10,000 TZM-bl cells (b) or PHA-stimulated PBMCs (c) were incubated for 48 to 72 h with 200 TCID_50_ of HIV-1_89.6_ and 10^8^ recombinant C. crescentus bacteria expressing GBVCE2 (Cc-GBVCE2). HIV-1 infection was measured using a β-galactosidase assay (TZM-bl cells) or p24 ELISA (PBMCs). To minimize assay-to-assay variability, the wells containing cells plus HIV-1 were set as 100% infection, and the results for the other wells were normalized to the 100% infection. An unpaired two-tailed *t* test was used to evaluate the significance of differences between groups. Each experiment was performed in quadruplicate and repeated three times. Results are reported as the mean + SEM. **, *P* < 0.01.

GB virus C (GBVC; human pegivirus, formerly hepatitis G virus) causes a persistent viral infection that does not cause any known disease pathology and appears to improve the survival of HIV-positive individuals and delay progression to AIDS (31–33). While many factors have been linked to this favorable survival (34, 35), the E2 protein of GB virus C (GBVCE2) is a putative fusion peptide that can interfere with HIV binding or fusion (36, 37). The E2 protein of GB virus C was expressed in the S layer of C. crescentus (Fig. 2a) and used in *in vitro* viral blocking assays with both TZM-bl cells and PBMCs (Fig. 2b and c). The E2 protein was successfully expressed in the S layer of C. crescentus, and recombinant C. crescentus bacteria expressing GBVCE2 (Cc-GBVCE2) were able to provide 57% protection from HIV-1_89.6_ in TZM-bl cells and 54% protection in PBMCs (Fig. 2). Furthermore, when Cc-GBVCE2 was tested for the ability to prevent HIV-1 infection with additional viral strains, it provided an average 65% reduction in HIV-1 infection in the TZM-bl cell line and 76% in PBMCs. These results suggest that Cc-GBVCE2 is an excellent candidate for additional microbicide testing.

### Cc-griffithsin, Cc-GBVCE2, Cc-elafin, and Cc-A1AT provide significant protection from HIV-1 infection in BLT mice.

**FIG 3:**
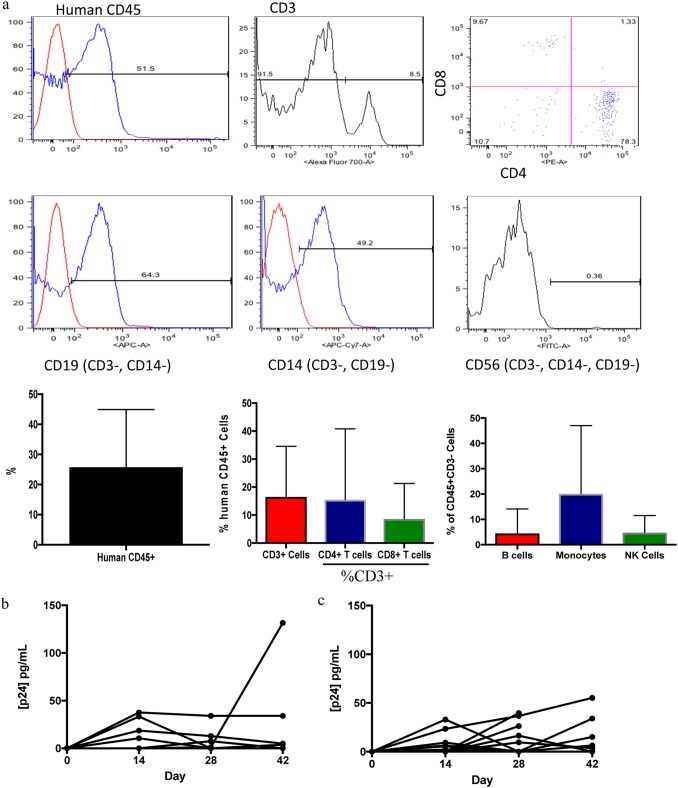
Humanized BLT mice. (a) Flow cytometry analysis of human immune cells in BLT mice. Bar plots are the mean + SD. (b) BLT mice were infected intravaginally with 10,000 TCID_50_ of HIV-1_JR-CSF_, and blood was analyzed by p24 ELISA. Data are for 17 mice. (c) BLT mice were infected intravaginally with 10,000 TCID_50_ of HIV-1_JR-CSF_ and 10^8^ Cc-control bacteria, and blood was analyzed by p24 ELISA. Data are for 15 mice.

These *in vitro* studies suggested that recombinant C. crescentus could be an excellent option for an HIV-1-specific microbicide and that further preclinical testing was warranted. Therefore, humanized BLT mice (28–30) were used to test the ability of the recombinant C. crescentus bacteria to provide protection from vaginal infection with HIV-1. Human immune cell reconstitution of BLT mice was confirmed by flow cytometry (Fig. 3a; Table 2). Vaginal infection of the mice with HIV-1_JR-CSF_ in the presence of 10^8^ Cc-control bacteria indicated that C. crescentus did not impact susceptibility to HIV-1 infection (Fig. 3b and c). We have previously demonstrated by titration that 10^8^ recombinant C. crescentus bacteria provide significant protection from infection *in vitro* without causing nonspecific inhibition of infection and have demonstrated that this dose can provide significant protection from vaginal infection with herpes simplex virus 2 (HSV-2) in C57BL/6 mice (13).

**TABLE 2:** Summary of human immune cell reconstitution in BLT mouse cohorts

Cohort identifier^*a*^	No. of mice	Avg % of CD45 cells (human)
1	3	46.4
2	7	21.8
4	14	29.2
5	14	27.5
6	8	21.1
7	4	71.5
8	14	47.3
9	19	39.2
10	14	16.6
11	10	32.4
12	16	17.4
13	12	12.9
14	10	—^*b*^
15	7	2.2
16	14	6.0
17	15	3.8

a Cohort 3 had 1 female mouse that was used as a control treated with PBS only.

b —, cohort 14 had an antibody staining problem during flow cytometry, but control mice only infected with HIV-1_JR-CSF_ had sustained vaginal infection, so microbicide experiments proceeded as normal for these mice.

While human immune cell reconstitution was variable across different cohorts of BLT mice, each cohort contained mice infected with HIV-1 only and mice infected with HIV-1 and treated with Cc-control, both groups of which were measured to be HIV-1 positive following infection, suggesting that each cohort was susceptible to vaginal infection with HIV-1. Furthermore, each recombinant C. crescentus bacterium was tested in multiple cohorts of BLT mice to verify that protection from HIV-1 infection was observed across more than one cohort.

**FIG 4:**
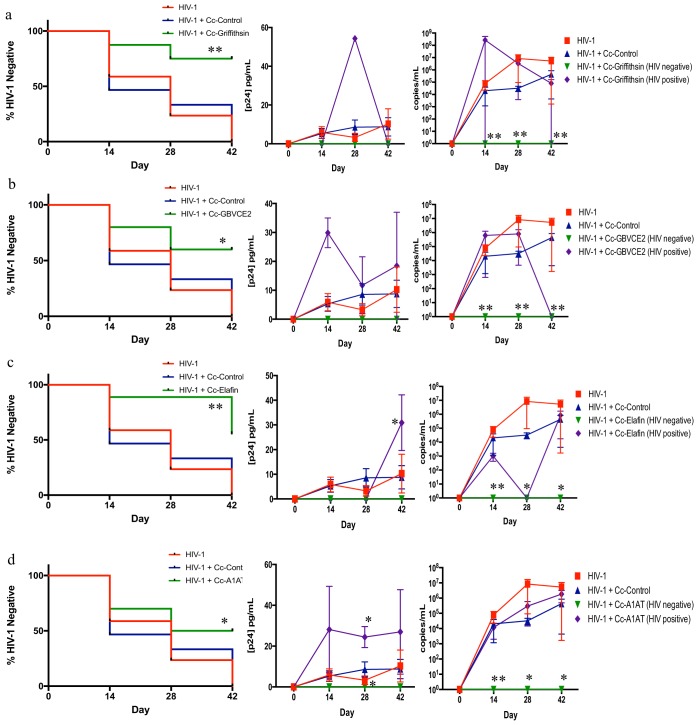
Recombinant C. crescentus bacteria protect from vaginal infection with HIV-1_JR-CSF_ in BLT mice. Humanized BLT mice were infected intravaginally with 10,000 TCID_50_ of HIV-1_JR-CSF_ in the presence or absence of 10^8^ recombinant C. crescentus bacteria. Blood was collected from the mice biweekly for 6 weeks and analyzed by p24 ELISA and RT-qPCR. Data are presented as a modified survival curve indicating when mice seroconverted, and statistics were performed as a log-rank test comparing HIV-1-infected mice receiving Cc-control and HIV-1-infected mice receiving a recombinant C. crescentus bacterium. p24 ELISA and RT-qPCR data are presented as the mean + SEM, and the presented statistics were performed by the Kruskal-Wallis test and represent the comparison between HIV-1-infected mice receiving Cc-control and HIV-1-infected mice receiving a recombinant C. crescentus bacterium. *, *P* < 0.05; **, *P* < 0.01. (a) Cc-griffithsin (*n* = 8); (b) Cc-GBVCE2 (*n* = 10); (c) Cc-elafin (*n* = 9); (d) Cc-A1AT (*n* = 10).

**FIG 5:**
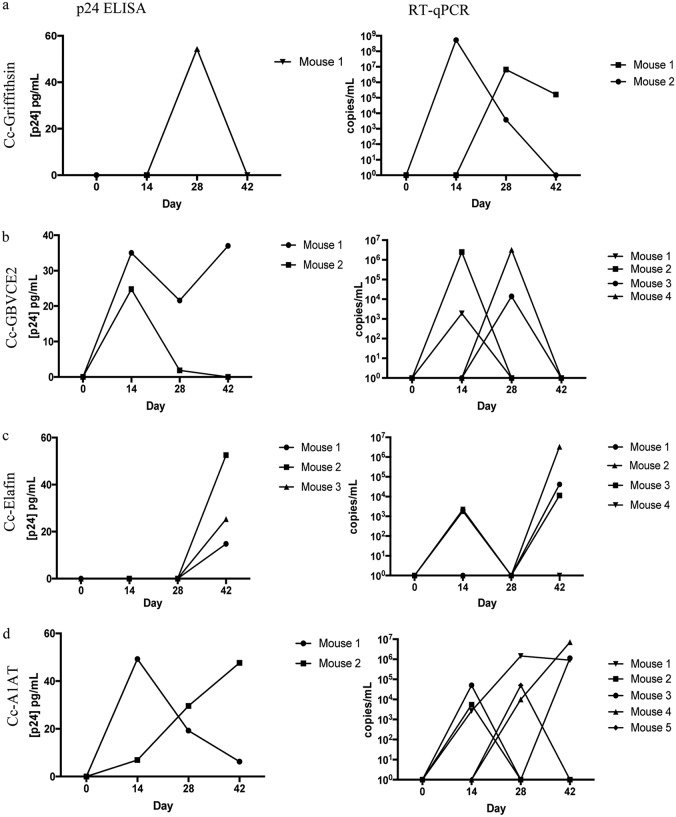
Individual viral loads in HIV-1-positive BLT mice. BLT mice were infected intravaginally with HIV-1_JR-CSF_ in the presence of 10^8^ recombinant C. crescentus bacteria, and HIV-1 infection levels were measured biweekly by p24 ELISA and RT-qPCR. Results are shown for each individual mouse that was measured to be HIV-1 positive. Mouse identifiers for p24 ELISA and RT-qPCR results are matched for each recombinant C. crescentus bacterium when a mouse was measured to be HIV-1 positive by both techniques. (a) Cc-griffithsin; (b) Cc-GBVCE2; (c) Cc-elafin; (d) Cc-A1AT.

**FIG 6:**
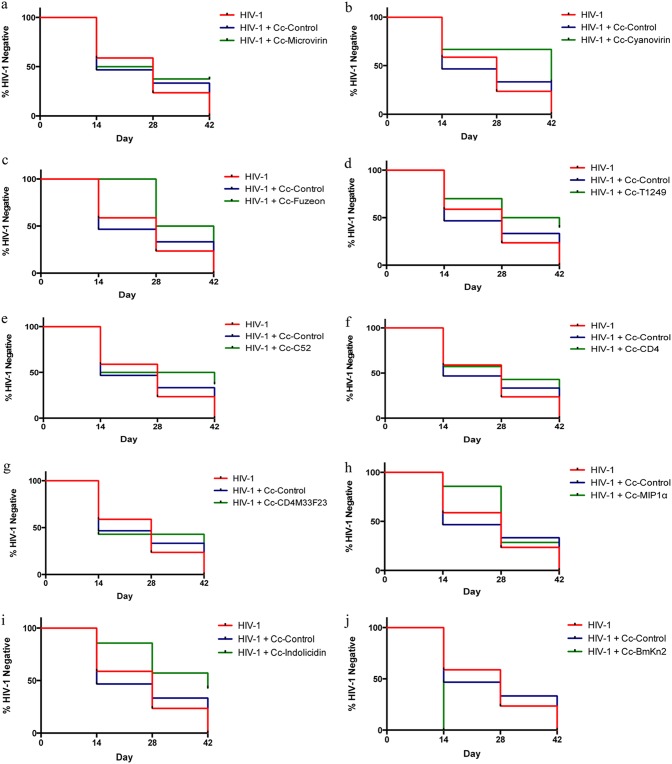
Recombinant C. crescentus in BLT mice. BLT mice were infected intravaginally with 10,000 TCID_50_ of HIV-1_JR-CSF_ in the presence or absence of 10^8^ recombinant C. crescentus bacteria. Data are presented as a modified survival curve indicating when the mice were measured to be HIV positive, and statistics were performed as a log-rank test comparing HIV-1-infected mice receiving Cc-control and HIV-1-infected mice receiving a recombinant C. crescentus bacterium. (a) Cc-microvirin (*n* = 8); (b) Cc-cyanovirin (*n* = 3); (c) Cc-Fuzeon (*n* = 8); (d) Cc-T1249 (*n* = 10); (e) Cc-C52 (*n* = 8); (f) Cc-CD4 (*n* = 7); (g) Cc-CD4M33F23 (*n* = 7); (h) Cc-MIP1α (*n* = 7); (i) Cc-indolicidin (*n* = 7); (j) Cc-BmKn2 (*n* = 3).

Cc-griffithsin was the most effective recombinant bacterium, protecting 6 of 8 mice (75%, *P* = 0.003) from vaginal infection with HIV-1, confirmed by both a p24 enzyme-linked immunosorbent assay (ELISA) and reverse transcription-quantitative PCR (RT-qPCR) (Fig. 4a and 5a). To ensure that all HIV-1-positive mice were detected, HIV-1 infection was measured by both p24 ELISA and RT-qPCR for each mouse, and a mouse was considered HIV-1 negative only if both assays had no virus detected. Interestingly, one mouse was HIV-1 negative by the p24 ELISA but had detectable HIV-1 RNA by RT-qPCR. Although the p24 levels in the blood of HIV-1-negative mice that received HIV-1 plus Cc-griffithsin (0 pg/ml p24) were not statistically significantly different from those of HIV-1-negative mice that received HIV-1 plus Cc-control, there was a significant reduction in HIV-1 RNA in the blood of mice that received HIV-1 plus Cc-griffithsin and that were HIV-1 negative compared to that in the blood of mice that received HIV-1 plus Cc-control. While the other antiviral lectins provided protection levels similar to those provided by Cc-griffithsin *in vitro*, recombinant C. crescentus bacteria expressing microvirin (Cc-microvirin) and cyanovirin (Cc-cyanovirin) were not effective at preventing infection in BLT mice. Cc-microvirin protected 37.5% (3 of 8, *P* > 0.05) of the BLT mice from HIV-1 infection (Fig. 6a). In a pilot experiment, Cc-cyanovirin protected 1 of 3 BLT mice from vaginal infection with HIV-1 (Fig. 6b). As this protection level was not statistically significant and at some times postinfection the HIV-1 levels were higher in mice that received HIV-1 plus Cc-cyanovirin, additional experiments were not undertaken.

Three additional recombinants were able to provide significant protection from vaginal infection in BLT mice: Cc-GBVCE2, recombinant C. crescentus bacteria expressing elafin (Cc-elafin), and recombinant C. crescentus bacteria expressing α-1-antitrypsin (Cc-A1AT). There were undetectable viral loads in 6 of 10 mice (60%, *P* = 0.0124) receiving Cc-GBVCE2 by both p24 ELISA and RT-qPCR following vaginal infection with HIV-1 (Fig. 4b and 5b). Cc-elafin provided 56% protection (*P* = 0.0036) from vaginal infection with HIV-1, with five of nine mice remaining HIV-1 negative, as determined by a lack of detection of p24 or viral RNA (Fig. 4c and 5c). Three mice that received Cc-elafin and that became HIV-1 positive had a delay in detectable levels of p24 in the blood until day 42 postinfection, although at this time p24 levels were significantly higher than the peak levels reached with HIV-1 alone. Cc-A1AT was able to protect 5 of 10 mice (50%, *P* = 0.0487) from vaginal infection with HIV-1, based on a lack of detection of p24 and viral RNA (Fig. 4d and 5d). Interestingly, those mice that received HIV-1 plus Cc-A1AT and that became HIV-1 positive appeared to have higher levels of virus in the blood, based on p24 levels.

Eight of the recombinant C. crescentus bacteria (those expressing Fuzeon [Cc-Fuzeon], T1249 [Cc-T1249], C52 [Cc-C52], CD4 [Cc-CD4], CD4M33F23 [Cc-CD4M33F23], MIP-1α [Cc-MIP1α], indolicidin [Cc-indolicidin], and BmKn2 [Cc-BmKn2]) provided some level of protection from vaginal infection with HIV-1, based on both p24 ELISA and RT-qPCR results, although this protection was not statistically significant (Fig. 6c to j).

## DISCUSSION

In this preclinical microbicide study, recombinant bacteria expressing the antiviral lectin griffithsin (Cc-griffithsin) were able to protect 75% of BLT mice from vaginal infection with HIV-1. Based on these results and the promising safety profile of griffithsin (16, 38), Cc-griffithsin represents an excellent option for additional testing as a potential HIV-1 microbicide.

Cc-GBVCE2 is another promising microbicide candidate, as it was able to protect 60% of BLT mice from vaginal infection with HIV-1. While these studies focused on the use of Cc-GBVCE2 as a topical agent to prevent HIV-1 transmission, it is possible that Cc-GBVCE2 may also have utility as a therapeutic option for HIV-1 infection. It has been reported that people coinfected with GB virus C and HIV-1 have a better prognosis and slower progression to AIDS, suggesting that GB virus C has beneficial effects on HIV-1 disease through multiple mechanisms (34, 35, 39–42), including the E2 portion of GB virus C being a putative fusion peptide that interferes with HIV binding or fusion and that prevents cell-to-cell spread (42–44). As such, Cc-GBVCE2 may be able to limit the spread of HIV-1 in infected individuals, as well as lower viral shedding to prevent transmission.

Interestingly, some mice appeared to be protected from HIV-1 infection initially but were measured to be HIV-1 positive at later time points. Further studies are necessary to investigate the potential mechanism responsible for this. Furthermore, some mice that became HIV-1 positive after receiving a recombinant C. crescentus bacterium that provided significant protection from HIV-1 infection in other mice appeared to have higher levels of HIV-1 infection than control mice, which may be a result of the variability of immune reconstitution inherent in this model.

Three of the microbicide candidates that were able to provide significant protection from vaginal infection with HIV-1 also target HSV-2: Cc-griffithsin, Cc-elafin, and Cc-A1AT (13, 45, 46). HSV-2 infection is a major risk factor for HIV-1 acquisition, increasing the risk of HIV acquisition by 2- to 4-fold (47–49). As HSV-2 can increase HIV-1 acquisition, this suggests that these recombinant C. crescentus bacteria could have a significant impact on HIV-1 infection rates, not only directly by preventing HIV-1 infection but also by preventing HSV-2 acquisition. In particular, Cc-A1AT provided 50% protection from HIV-1 infection in BLT mice and provided an 86% increase in survival following vaginal infection in C56BL/6 mice, whereas Cc-griffithsin provided 75% protection from HIV-1 infection and 57% protection from HSV-2 disease (13).

While these studies investigated using the recombinant C. crescentus individually, it is likely that combining the successful recombinant C. crescentus candidates will increase HIV-1 protection beyond those levels observed with each one individually. In addition, as all recombinant C. crescentus bacteria provided at least some low-level protection from HIV-1 infection, it is possible that combining multiple candidates that target different aspects of the HIV-1 attachment and entry process, including those that did not significantly reduce HIV-1 acquisition in BLT mice, may generate the most effective microbicide cocktail. In support of this, we have previously demonstrated that combining Cc-CD4 and Cc-MIP1α *in vitro* increases HIV-1 protection from 50 to 75% to 97% (11).

Our studies have focused on investigating a dosing strategy that would be coitally dependent, similar to a vaginal gel, as the first phase of testing. As a coitally dependent option may not be feasible in a real-world situation, we have begun to undertake additional studies to improve the utility of a C. crescentus-based microbicide. Preliminary data indicate that C. crescentus is able to prevent infection up to 8 h after application, and additional studies are ongoing to determine if this can be extended further. In addition, preliminary studies in an HSV-2 model suggest that C. crescentus may have some ability to prevent infection when applied after exposure. We have not investigated the use of recombinant C. crescentus as a long-term prevention option, such as on a vaginal ring, but it should be possible to assemble the recombinant S-layer proteins on a vaginal ring to provide long-term HIV-1 prevention. While C. crescentus is a nonpathogenic bacterium found in freshwater sources, including drinking water in the United States (50), the possibility does exist for immune responses to develop after prolonged usage. Our previous studies support a lack of immune response after the short-term use of C. crescentus in mouse models (13, 27, 51). In particular, the lipopolysaccharide (LPS) of C. crescentus has been found to produce greater than 100-fold less tumor necrosis factor than LPS isolated from Escherichia coli (51). To minimize the risk of an immune response, creating an abiotic delivery system containing just the recombinant S-layer proteins would greatly reduce the chance of an immune response.

HIV-1 remains a major global health priority, particularly for young women. A recombinant C. crescentus-based microbicide represents a safe and cost-effective option that should undergo further investigation for female-controlled prevention of HIV-1 as well as HSV-2. If a C. crescentus microbicide is developed, it could be combined with other prevention strategies for sexually transmitted infections and pregnancy as part of a comprehensive reproductive health package. In addition, adding a successful microbicide to the current HIV prevention tool kit will fulfill the need for a discreet, easy-to-use product that is targeted for women and that does not require male consent or continuous prophylactic antiretroviral use. Further preclinical studies are warranted to continue the investigation of a recombinant C. crescentus-based microbicide in a nonhuman primate model or clinical setting.

## MATERIALS AND METHODS

### Bacterial strains and growth conditions.

Recombinant C. crescentus strain JS4038 was grown in PYE medium (0.2% peptone, 0.1% yeast extract, 0.01% CaCl_2_, 0.02% MgSO_4_) with 2 μg/ml chloramphenicol at 30°C. Gene segments were synthesized by Integrated DNA Technologies, Inc. (Coralville, IA), with codon usage being adapted for C. crescentus. C. crescentus bacteria displaying chimeric S-layer proteins have been previously described (11–13). The amino acid sequence for Cc-GBVCE2 is WDRGNVTLLCDCPNGPWVWVPAFCQAVG. The synthesized DNA segment also specified BglII and SpeI restriction sites on the 5′ side and an NheI site on the 3′ end to facilitate directional cloning into p4BRsaA(723)/GSCC digested with BglII and NheI (52, 53).

### Preparation of C. crescentus cells.

C. crescentus S-layer display constructs were grown in PYE medium to an optical density at 600 nm of approximately 1 (3.1 × 10^9^ cells/ml). Cells were centrifuged and suspended in sterile water. This was repeated one time for *in vitro* experiments and three times for *in vivo* experiments.

### Cell lines.

293T cells were a gift from Ninan Abraham (University of British Columbia) and were maintained in Dulbecco’s modified Eagle medium (DMEM) with 7.5% fetal bovine serum (FBS), 100 U/ml penicillin, and 0.1 mg/ml streptomycin. TZM-bl cells were obtained through the NIH AIDS Reagent Program, Division of AIDS, NIAID, NIH, and maintained in DMEM with 7.5% FBS (Gibco), 100 U/ml penicillin, and 0.1 mg/ml streptomycin (Gibco) as previously described (13). 174xCEM cells were obtained through the NIH AIDS Reagent Program, Division of AIDS, NIAID, NIH, and maintained in RPMI 1640 medium supplemented with 10% FBS as previously described (13).

### Primary cells.

Whole blood was collected and processed using Lymphoprep medium (Stemcell Technologies Inc.). PBMCs were grown in RPMI 1640 supplemented with 20% FBS.

### HIV-1.

The following reagents were obtained through the NIH AIDS Reagent Program, Division of AIDS, NIAID, NIH: HIV-1_89.6_ was from Ronald Collman (54); pYK-JRCSF (catalog number 2708) was from Irvin S. Y. Chen and Yoshio Koyanagi (55–57) and was a gift from Zabrina Brumme (Simon Fraser University); HIV-1_SF162_ was from Jay Levy (58); HIV-1_BaL_ was from Suzanne Gartner, Mikulas Popovic, and Robert Gallo (59, 60); and HIV-1_JR-FL_ was from Irvin S. Y. Chen (56, 61, 62).

### HIV-1 propagation.

HIV-1_89.6_ was propagated in 174xCEM cells with 7.5 μg/ml DEAE-dextran (Sigma) and was harvested at the peak cytopathic effect. HIV-1_pykJR-CSF_ was propagated in 293T cells using the Lipofectamine 2000 reagent (Invitrogen) according to the manufacturer’s instructions. HIV-1_JR-FL_ and HIV-1_SF162_ were propagated in 5 × 10^6^ phytohemagglutinin (PHA)-stimulated PBMCs using 10 μg/ml hexadimethrine bromide (Polybrene; Sigma). HIV-1_BaL_ was propagated in PHA-stimulated PBMCs with 7.5 μg/ml DEAE-dextran.

### Virus titration.

Serial dilutions of virus were prepared in 96-well plates using medium containing 7.5 μg/ml DEAE-dextran. TZM-bl cells (10,000) were added. The plates were maintained at 37°C in 5% CO_2_ for 48 h. Infection of cells was measured indirectly using a mammalian β-galactosidase assay kit (Pierce), followed by reading of the absorbance at 415 nm. An absorbance of greater than 0.2 was considered a positive infection. The 50% tissue culture infectious dose (TCID_50_) per milliliter was determined for each viral stock by identifying the dilution of virus in which 50% of the TZM-bl cells were infected.

### Virus blocking experiments.

The C. crescentus constructs were grown and prepared as described above. Experiments were carried out in quadruplicate wells of 96-well plates. The volume of virus added was determined by calculating the 200 TCID_50_ value, and 1 × 10^8^ C. crescentus cells were added to each well. The virus and C. crescentus constructs were incubated for 1 h at 37°C before adding 10,000 TZM-bl cells or PBMCs and 7.5μg/ml DEAE-dextran to each well. Cc-MIP1α was incubated with the TZM-bl cells or PBMCs for 1 h before virus addition. The level of infection was determined after 48 to 72 h by use of a β-galactosidase assay kit (TZM-bl cells) or a p24 ELISA (ZeptoMetrix Corporation and ProSci Incorporated) according to the manufacturers’ instructions (PMBCs). For the β-galactosidase assays, the data are presented and determined as a percentage of infection of the wells with TZM-bl plus HIV-1 with the background from uninfected TZM-bl cells subtracted. p24 ELISA data were normalized to infection of the untreated control wells with PBMCs and HIV-1, set as 100%.

### Preparation of humanized bone marrow-liver-thymus (BLT) mice.

NSG mice were obtained from The Jackson Laboratory and maintained in the Modified Barrier Facility at the University of British Columbia. Six- to 12-week-old female mice were used in the experiments. Fetal liver was split, and 1-mm^3^ pieces of liver and thymus were implanted under the kidney capsule. Autologous fetal liver tissue was used for CD34^+^ cell isolation using a CD34^+^ cell positive selection kit from Stemcell Technology Inc. according to the manufacturer’s instructions, and cells were frozen in 90% human serum–10% dimethyl sulfoxide. At 3 weeks following the surgical implantation, mice received 225 cGy of irradiation from an X-ray source, followed by intravenous injection of 100,000 to 200,000 CD34^+^ cells within 30 h. The mice were incubated for 9 weeks before blood was characterized for human immune cell reconstitution.

### Flow cytometry.

One hundred microliters of blood was collected from BLT mice and treated with EDTA. Red blood cell lysis buffer was added, and samples were incubated for 15 min before washing with fluorescence-activated cells sorting buffer (phosphate-buffered saline [PBS] plus 2% FBS). Cells were stained with anti-mouse CD45 Pacific Blue (eBioscience), mouse anti-human CD45 phycoerythrin (PE)-Cy7 (BD Pharmingen), mouse anti-human CD3 Alexa Fluor 700 (eBioscience), mouse anti-human CD4 PE (BD Pharmingen), mouse anti-human CD8 PE-Cy5 (BD Pharmingen), mouse anti-human CD14 allophycocyanin (APC)-Cy7 (BD Pharmingen), mouse anti-human CD19 APC (BD Pharmingen), and mouse anti-human CD56 fluorescein isothiocyanate (FITC) (BD Pharmingen). Data were acquired on an LSR II flow cytometer and analyzed using FlowJo software (TreeStar). Mouse versus human CD45^+^ cells were examined to exclude any mouse CD45^+^ cells and include human CD45^+^ cells. The human CD45^+^ cells were gated for expression of CD3. CD3^+^ cells were examined for expression of CD4 and CD8. The CD3^−^ cells were examined for expression of CD19 (B cells) versus CD56 (NK cells). Unstained and single-stained human PBMCs were used as controls.

### HIV-1_JR-CSF_ infection.

Mice were anesthetized before atraumatic vaginal infection with 10,000 TCID_50_ of HIV-1_JR-CSF_ in the presence or absence of 10^8^ C. crescentus bacteria. The C. crescentus bacteria were premixed with HIV-1_JR-CSF_ less than 5 min before inoculation into the vaginal tract in a volume of 20 μl using a sterile p200 pipette tip, and then the entire volume was inoculated intravaginally. Sixteen independent cohorts of BLT mice were created for these studies. Each cohort contained at least one mouse infected with HIV-1_JR-CSF_ only and one mouse infected with HIV-1_JR-CSF_ and treated with Cc-control. Each recombinant C. crescentus bacterium was tested in mice from at least 2 different cohorts.

### HIV-1_JR-CSF_ infection analysis.

Blood was collected from the retro-orbital sinus on days 0, 14, 28, and 42 postinfection. Blood was centrifuged at 8,000 rpm for 13 min, and serum was aliquoted and frozen at −80°C before being analyzed by p24 ELISA (ZeptoMetrix) and RT-qPCR. The p24 ELISA was performed according to the manufacturer’s instructions and had a limit of detection of 3.9 pg/ml. Viral RNA was extracted from equal volumes of serum using a QIAamp viral RNA minikit (Qiagen). Equal volumes of RNA were converted to cDNA using an Applied Biosystems cDNA kit (Thermo Fisher Scientific). Equal volumes of cDNA were run in duplicate on an Mx3005p PCR multiplex quantitative PCR instrument (Stratagene) with Sybr green (Bio-Rad), forward primer ATCAAGCAGCTATGCAAATGCT, and reverse primer CTGAAGGGTACTAGTAGTCCCTGCTATGTC; the settings were 50°C for 2 min, 95°C for 10 min, and 40 cycles at 95°C for 15 s and 60°C for 1 min. The limit of detection was 500 copies/ml. Mice were considered HIV-1 negative if HIV-1 was not detected by both the p24 assay and the RT-qPCR at all time points.

### Statistics.

Statistical analysis was performed with GraphPad Prism software. Viral blocking assay results are reported as the mean + standard error of the mean (SEM). Experiments were conducted in quadruplicate wells and repeated in a minimum of three independent experiments, unless indicated otherwise. An unpaired two-tailed *t* test (for comparison of the results for mice receiving the Cc-control and those receiving 1 recombinant C. crescentus bacterium) or analysis of variance (ANOVA) with Bonferroni’s correction for multiple comparisons (for comparison of the results for mice receiving the Cc-control and >2 groups receiving recombinant C. crescentus bacteria) was used as appropriate to evaluate the significance of differences between groups. Statistical analysis for BLT mice was performed using the log-rank (Mantel-Cox) test for survival curves and the Kruskal-Wallis test with Dunn’s multiple-comparison test for p24 ELISA and RT-qPCR analysis. The reported statistics represent the comparison between mice infected with HIV and receiving Cc-control and mice infected with HIV and receiving a recombinant C. crescentus bacterium. A *P* value of <0.05 was considered statistically significant.

### Study approval.

All animal work was approved by the University of British Columbia Animal Care Committee (protocols A13-0055, A13-0234, and A12-0245). Human ethics approval was obtained from the University of British Columbia Clinical Ethics Board (H12-02480). Human PBMCs were obtained from healthy donors after they provided informed written consent. Second-trimester human fetal liver and thymus tissue was obtained after informed written consent from women undergoing elective abortion and procured by Advanced Biosciences Resources Inc. (Alameda, CA).
